# Metabolomic Investigation of β-Thalassemia in Chorionic Villi Samples

**DOI:** 10.3390/jcm8060798

**Published:** 2019-06-05

**Authors:** Giovanni Monni, Federica Murgia, Valentina Corda, Cristina Peddes, Ambra Iuculano, Laura Tronci, Antonella Balsamo, Luigi Atzori

**Affiliations:** 1 Department of Prenatal and Preimplantation Genetic Diagnosis and Fetal Therapy, Ospedale Pediatrico Microcitemico “A.Cao”, 09121 Cagliari, Italy; cordavale@hotmail.it (V.C.); cristina.peddes@gmail.com (C.P.); ambraiuculano76@gmail.com (A.I.); 2 Department of Biomedical Sciences, Clinical Metabolomics Unit, University of Cagliari, 09121 Cagliari, Italy; federica.murgia@unica.it (F.M.); lauratronci90@gmail.com (L.T.); antonella.balsamo1@teletu.it (A.B.); latzori@unica.it (L.A.)

**Keywords:** β-thalassemia, chorionic villi samples, metabolomics, gas chromatography–mass spectrometry, pentose phosphate pathway, arachidonic acid metabolism

## Abstract

Background: Beta-thalassemias are blood disorders characterized by poorly understood clinical phenotypes ranging from asymptomatic to severe anemia. Metabolic composition of the human placenta could be affected by the presence of pathological states such as β-thalassemia. The aim of our study was to describe metabolic changes in chorionic villi samples of fetuses affected by β-thalassemia compared to a control group by applying a metabolomics approach. Methods: Chorionic villi samples were differentiated according to the genetic diagnosis of β-thalassemia: control (Group 1, *n* = 27); heterozygous (Group 2, *n* = 7); homozygous (Group 3, *n* = 7). Gas chromatography–mass spectrometry was used to detect the metabolic composition of the samples. Subsequently, multivariate and univariate statistical analysis was performed. The discriminant metabolites were used to identify the altered pathways. Results: Supervised multivariate models were devised to compare the groups. The model resulting from the comparison between Group 1 and Group 3 was the most significant. Discriminant metabolites were identified, and the most altered pathways were as follows: pentose phosphate pathway (PPP), arachidonic acid metabolism, glycolysis, and gluconeogenesis, suggesting the presence of an energetic shift toward the PPP and the presence of oxidative stress in β-thalassemia chorionic villi samples. Conclusions: The metabolomics approach identified a specific metabolic fingerprint in chorionic villi of fetuses affected by β-thalassemia.

## 1. Introduction

β-thalassemias are a group of hereditary blood disorders characterized by abnormal beta globin production [1]. These conditions result in a broad range of phenotypes, from severe anemia to clinically asymptomatic forms, and the mechanisms underlying the phenotypic heterogeneity of β-thalassemia are still poorly understood [2,3]. A better molecular understanding of thalassemias, the development of procedures for their detection by DNA analysis, and the introduction of chorionic villi sampling and preimplantation genetic diagnosis (PGD) have improved prenatal detection of these disorders [4,5].

During human placenta development, alterations in metabolite composition could be affected by the presence of pathological states. The metabolic profile of the placenta may improve our understanding of abnormal mechanisms such as β-thalassemia and could potentially allow for early phenotype prediction.

Metabolomics is one of the most powerful and promising tools for the analysis of placental metabolism [6,7,8]. Analytical techniques such as mass spectrometry (MS) can provide information about tissue metabolites including lipids, amino acids, and high-energy metabolites, thus providing an efficient method for monitoring altered pathways [9]. This could provide an integrated “snapshot” of the metabolic change during different conditions [10,11,12]. Several studies have been performed on the metabolic alterations in patients with β–thalassemia [13,14,15]; however, to our knowledge, the metabolic profile from chorionic villi of β-thalassemia fetuses collected after transabdominal chorionic villi sampling (TA-CVS) has not yet been evaluated.

The aim of our study was to characterize the metabolic changes in chorionic villi samples of fetuses affected by β-thalassemia compared to a control group.

## 2. Materials and Methods

Samples for this prospective study were collected from the Department of Obstetrics and Gynecology, Prenatal and Preimplantation Genetic Diagnosis, Fetal Therapy, Microcitemico Pediatric Hospital “A.Cao” in Cagliari, Sardinia, Italy. All women with fetuses at risk of β-thalassemia underwent non-directive genetic counseling and prenatal invasive diagnosis by TA-CVS [16]. After institutional review board approval, written consent was obtained from all participating women prior to performing TA-CVS. All chorionic villi sampling (CVS) procedures were performed between the 11th and the 14th weeks of pregnancy by free-hand transabdominal technique by a single operator (G.M.) [17]. After sampling, an adequate specimen of chorionic villi was used for cytogenetic examination, and the remaining aliquot was reserved for metabolomics analysis. It was frozen within 2 min in liquid nitrogen and kept at ‒80 °C.

Patient demographics (ethnic group and maternal age), ultrasound data (crown rump length (CRL) and fetal nuchal translucency (NT) measurement), and biochemical parameters (pregnancy-associated plasma protein A and free β-human chorionic gonadotropin) were collected in our database (Table 1).

Samples were divided into three groups based on the outcome of the genetic results: normal fetuses (Group 1, *n* = 27), heterozygous fetuses (Group 2, *n* = 7), and homozygous fetuses (Group 3, *n* = 7). The metabolomics profiles of Group 1, Group 2, and Group 3 are described in Table 1. The metabolic profiles of Group 2 and Group 3 were compared to Group 1. In the analyzed cohort, heterozygous and homozygous fetuses were characterized by the β cd39 (C > T) mutation.

### 2.1. Sample Preparation

CVS aliquots were analyzed as previously described [18]. The aliquots were mixed with 800 µL of methanol and 200 µL of Milli-Q water and then vortexed. After 30 min of sonication, samples were kept at ‒20 °C for 20 min and then centrifuged at 8600 g for 10 min at 4 °C. The supernatant was collected for analysis.

### 2.2. Gas Chromatography Mass–Spectrometry Analysis and Data Processing

For gas chromatography–mass spectrometry (GC–MS) analysis, 400 µL of each extract was dried with a vacuum concentrator overnight (Eppendorf concentrator plus, Eppendorf AG, Hamburg, Germany) and derivatized with 25 μL of methoxyamine dissolved in pyridine (10 mg/mL) (Sigma-Aldrich, St. Louis, MO, USA) at 70 °C. After 1 h, 50 μL of N-Methyl-N-(trimethylsilyl)-trifluoroacetamide (MSTFA, Sigma-Aldrich, St. Louis, MO, USA) was added, and the samples were left at room temperature for 1 h. Successively, samples were diluted in 50 μL of hexane (Sigma-Aldrich, St. Louis, MO, USA), and 1 μL of derivatized sample was injected splitless into a 7890A gas chromatograph coupled with a 5975C Network mass spectrometer (Agilent Technologies, Santa Clara, CA, USA) equipped with a 30 m × 0.25 mm internal diameter ID fused silica capillary column with a 0.25 μM TG-5MS stationary phase (Thermo Fisher Scientific, Waltham, MA, USA). The injector and transfer line temperatures were at 250 °C and 280 °C, respectively. The gas flow rate through the column was 1 mL/min. The column’s initial temperature was kept at 60 °C for 3 min, increased to 140 °C at 7 °C/min, held at 140 °C for 4 min, increased to 300 °C at 5 °C/min, and kept for 1 min. The identification of metabolites was performed using the standard NIST 08 and Golm Metabolome Database (GMD) mass spectra libraries, as well as by comparison with authentic standards, when available.

The R library XCMS [19,20] was used for peak detection and retention time correction. Parameters utilized for peak deconvolution for GC–MS matrices were manually optimized. The resulting matrices were processed using an in-house Python script to eliminate signals present in the blanks, keeping only the most abundant feature per molecule and modifying all zeros present in the matrix by inserting half of the minimum value found for a feature. After manual correction of the filtered matrix to eliminate the internal standard and any possible remaining noise signal, median fold change normalization was performed using an in-house Python script in order to compensate for sample dilution biases [21].

### 2.3. Statistical Analysis

A multivariate statistical analysis was performed on GC–MS data by using SIMCA-P software (ver. 15.0, Umetrics, Sweden) [22]. The variables were unit variance (UV) scaled. The initial data analyses were conducted using principal component analysis (PCA), which is important for the exploration of sample distributions without classification. To identify potential outliers, DmodX and Hotelling’s T2 tests were applied, and then, a supervised analysis was used. Partial least square discriminant analysis (PLS-DA) maximizes the discrimination between samples assigned to different classes. The variance and predictive ability (R^2^X, R^2^Y, Q^2^) were established to evaluate the suitability of the models. A permutation test (*n* = 400) was performed to validate the models. The scores from each PLS-DA model were subjected to a CV-ANOVA (*p* value < 0.05 was considered statistically significant). 

The most significant variables were extracted by the loadings plot from the PLS-DA model. GraphPad Prism software (version 7.01, GraphPad Software, Inc., San Diego, CA, USA) was used to perform the univariate statistical analysis of the data resulting from the multivariate analysis. To verify the significance of the resulting metabolites, a Mann–Whitney U test was performed.

### 2.4. Pathways Analysis

Metabolic pathways were generated by using MetaboAnalyst 3.0, a web server designed to obtain comprehensive metabolomic data analysis, visualization, and interpretation [23]. This approach permits correlation of metabolite changes with metabolic networks. The pathway analysis module of Metaboanalyst 3.0 combines results from pathway enrichment analysis with the pathway topology analysis to identify the most relevant pathways involved in the conditions under study. It uses high-quality KEGG metabolic pathways (Kyoto Encyclopedia of Genes and Genomes) as the backend knowledge base.

## 3. Results

**Figure 1:**
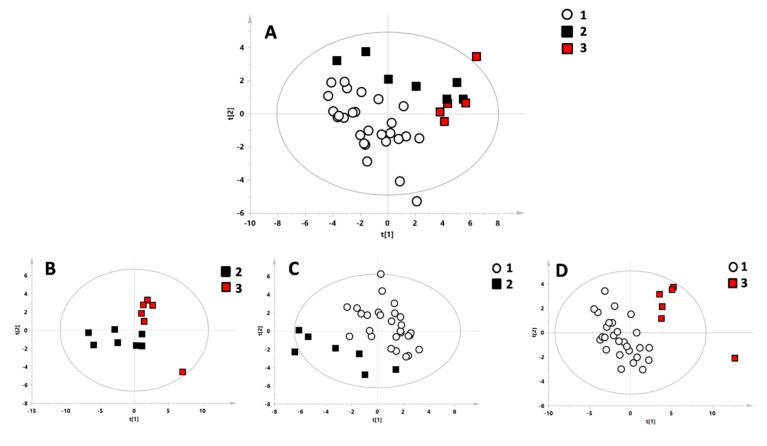
Multivariate statistical models of the different investigated classes. (**A**) Model with all three group: white circles = Group 1 (control), black boxes = Group 2 (heterozygous for β-thalassemia), and red boxes = Group 3 (homozygous for β-thalassemia). (**B**) Model comparing heterozygous and homozygous for β-thalassemia. (**C**) Model comparing the control group and heterozygous for β-thalassemia. (**D**) Model comparing the control group and homozygous for β-thalassemia.

Groups 1 (control), 2 (heterozygous), and 3 (homozygous) were compared through multivariate statistical analysis to highlight possible differences in their metabolic profiles. PCA models for all of the groups were constructed. One sample from Group 3 was identified as a strong outlier through the Hotelling T^2^ test and was excluded. Furthermore, a PLS-DA model with all three groups was created (Figure 1A). To identify specific features for each group, models were constructed comparing the single groups pairwise. The comparison between Group 1 and Group 2 (Figure 1C) was statistically significant (*p*-value < 0.05), as was the comparison between Group 1 and Group 3 (Figure 1D), while the comparison between Group 2 and Group 3 was not significant (Figure 1D). The models were validated with the respective permutation tests.

All the statistical parameters of the models are reported in Table 2.

**Figure 2:**
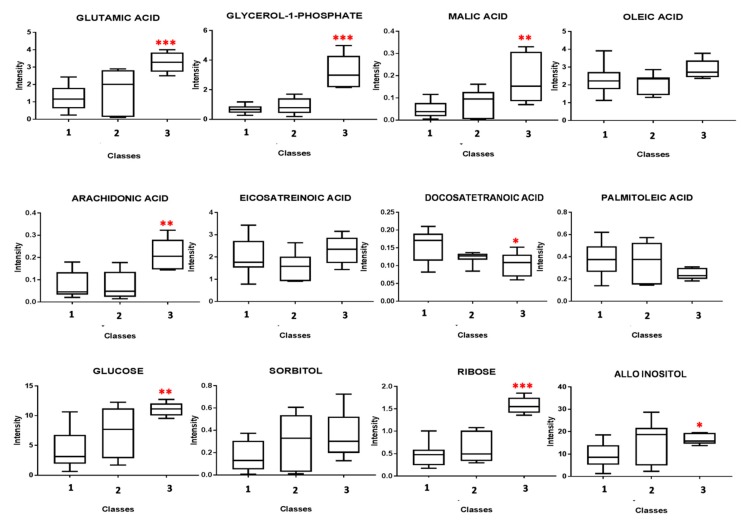
Box plots of the most altered metabolites resulting from the comparison between classes 1, 2, and 3. The average concentrations of the indicated metabolites underwent the Mann–Whitney U test. (* indicates a *p*-value < 0.05, ** indicates a *p*-value < 0.001, *** indicates a *p*-value < 0.0001).

Through analysis of the single models, it was possible to identify the metabolic fingerprint of each class. The most important metabolites were evaluated through analysis of the loadings plot. The resulting metabolites from the comparison between the groups underwent univariate analysis by using the Mann–Whitney U test to evaluate the p-value. Trends of the average concentrations of the most altered metabolites are reported as box plots in Figure 2.

**Figure 3:**
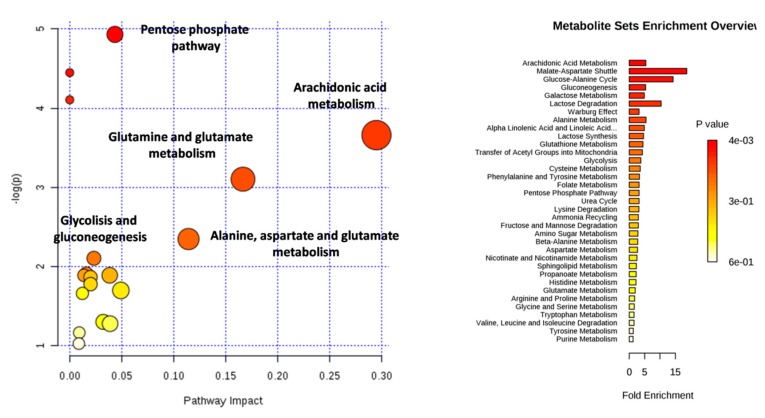
Altered pathways in chorionic villi samples affected by β-thalassemia resulting from the analysis with Metaboanalyst.

Glutamic acid, glycerol-1-phosphate, malic acid, arachidonic acid (ARA), glucose, and ribose were found to significantly increase in homozygous patients, while docosatetranoic acid and palmitoleic acid were found to decrease when compared with the heterozygous and the control group. Subsequently, pathway analysis was performed by using the web-based analytical tool Metaboanalyst 4.0. The pathways most altered in homozygous patients were as follows: pentose phosphate pathway (PPP), ARA metabolism, glutamine and glutamate metabolism, alanine, aspartate and glutamate metabolism, glycolysis, and gluconeogenesis (Figure 3).

## 4. Discussion

New innovative omics technologies offer the possibility to screen for novel biomarkers and to better understand pathological processes [15,24]. Metabolomics is a tool that can be used with this aim to investigate common and disabling genetic conditions such as β-thalassemia. Pathological and environmental stress conditions can change the expression levels of certain genes and, hence, the metabolite concentrations of the corresponding pathways. The phenotypic and genetic heterogeneity of β-thalassemia are challenging to study, especially in early gestation. In this study, the metabolome of chorionic villi was investigated to better understand the still unclear pathophysiological mechanisms of β-thalassemia. GC–MS analysis and multivariate statistics identified different metabolic profiles in the chorionic villi of fetuses affected by homozygous compared to heterozygous β-thalassemia and control fetuses. Glutamic acid, glycerol-1-phosphate, malic acid, ARA, glucose, and ribose were found to significantly increase in homozygous patients, while docosatetranoic acid and palmitoleic acid were found to decrease. PPP, ARA metabolism, glutamine and glutamate metabolism, alanine, aspartate and glutamate metabolism, glycolysis, and gluconeogenesis were potentially altered in both the homozygous and heterozygous groups.

PPP has two major functions: (1) Production of reduced nicotinamide adenine dinucleotide phosphate (NADPH), which is used as a reducing agent in several biosynthetic pathways and is also important for protection against oxidative damage. (2) synthesis of ribose 5-phosphate, which is required for nucleotide and nucleic acid synthesis [25]. Considering the tissue distribution in humans, the expression levels of PPP enzymes vary widely from tissue to tissue. Relatively high levels are found in the liver, the adrenal cortex, the testicles and ovaries, the thyroid, and the blood in the red blood cells. In all of these, a continuous supply of NADPH is required to support reductive biosynthesis and/or to counteract the effects of oxygen free radicals or reactive oxygen species (ROS). Contrasting action on the effects of ROS is particularly important in cells such as red blood cells, which are directly exposed to oxygen [26]. In erythrocytes, the main function of PPP appears to be the maintenance of glutathione in the reduced state and preservation of cell membrane structure [27]. NADPH is used for the reduction of oxidized glutathione (GSSG) to reduced glutathione (GSH). GSH is important for the detoxification of ROS and converts reactive hydrogen peroxide into H_2_O. Moreover, high levels of enzymes of this metabolic pathway are also present in rapidly dividing cells such as those of the embryo in the early stages of development. The alteration of this pathway in CVS samples with β-thalassemia suggests both a demand of high quantities of ribose 5-phosphate for nucleotide synthesis (typical of embryonic development) and increased oxidative stress with the need to respond with anti-oxidative mechanisms such as NADPH production and PPP activation (particularly considering the comparison with the control group). Oxidative stress is an important mechanism in the progression of β-thalassemia, and alterations in this pathway are not unexpected. Oxidative stress in patients with β-thalassemia is mainly caused by peroxidative injury due to secondary iron overload. Additionally, production of free radicals by iron overload plays an important role in the pathogenesis of β-thalassemia [28]. Moreover, the oxidation of hemoglobin alpha leads to the formation of hemicromes. Hemicromes bind to and modify many components of the red blood cell membrane precipitating and causing the disintegration of the heme, resulting in the release of compounds containing toxic iron. Free iron catalyzes the formation of active oxygen compounds [29].

In patients with β-thalassemia, antioxidant molecules and enzymes are significantly decreased. The continuous production of ROS overwhelms the antioxidant machinery very rapidly [30]. Oxidant damage to thalassemic erythroid precursors can cause their accelerated apoptosis and ineffective erythropoiesis [31]. One of the best described effects of ROS on cells is the oxidative modification of fatty acids within membrane phospholipids, i.e., lipoperoxidation [32]. Oxidative modification of membrane phospholipids can result in the alteration of membrane fluidity, protein structure, and cell signaling. Oxidative stress and peroxidation of membrane phospholipids have been shown to enhance phospholipases A2 (PLA2) activity [33], which can give rise to a number of biologically active mediators such as ARA, a polyunsaturated fatty acid present in the phospholipids of membranes of the body’s cells. The major action of ARA is the promotion of acute inflammatory response, characterized by the production of proinflammatory mediators [34] such as PGE_2_ and PGI_2_. The study of β-thalassemia is not simple, especially from a metabolic point of view and in an early stage such as embryonic life. The interpretation of the metabolic changes in fetuses affected by β-thalassemia requires a strong effort considering both the lack of information in scientific literature and the heterogeneity of this disease; therefore, our findings can only provide a hypothesis. Fetal hemoglobin is formed by one alpha and two gamma subunits. These fetuses have the gene mutation for the beta globin, which is the only variable that differentiates them from the control group in our analysis. We can hypothesize that the presence of oxidative stress could be correlated to the presence of the mutation even if the defective beta globin has not yet been expressed.

## 5. Conclusions

In this study, an increase in the metabolites involved in energetic pathways, such as the pentose phosphate pathway, directly linked to the production of antioxidant species, was observed in chorionic villi from fetuses with homozygous β-thalassemia, suggesting an oxidative stress status. Oxidative stress negatively impacts several biological functions of β-thalassemia and can play an important role in its pathogenesis. A better understanding of the primary biological events of this condition may provide new avenues for the management of this serious condition.

This was a preliminary study that showed how metabolic alterations could be present in early pregnancy in placental tissue of fetuses affected by β-thalassemia. Due to the novelty of these results and considering the lack of studies of metabolism changes in β-thalassemia patients, further investigation is necessary.

## Figures and Tables

**Table 1:** Data of the patients enrolled in the study. (Average maternal age, gestational age, nuchal translucency, and crown-rump length).

	Patients (*n*)	Age (years)	Gestational Age (weeks)	NT (mm)	CRL (mm)
**Control**	27	36.8 ± 5.3	12.72 ± 0.75	2.07 ± 0.9	61.34 ± 9.8
**Heterozygotes**	7	31.2 ± 4.1	12.2 ± 1.8	1.4 ± 0.4	53.0 ± 5.4
**Homozygotes**	6	34.7 ± 2.9	11.9 ± 0.55	1.5 ± 0.3	52.9 ± 5.2

*n*: number of Patients; nuchal translucency (NT); crown-rump length (CRL); data are mean ± standard deviation.

**Table 2:** Summary of the statistical parameters of the multivariate models.

	Supervised Models					
	*N*	R^2^X	R^2^Y	Q^2^	*p*-Value	Permutation Test: Intercept R^2^\Q^2^
**C vs. Hom vs. Het**	39	0.351	0.484	0.159	ns	0.27/−0.14
**Het vs. Hom**	13	0.515	0.699	0.246	ns	0.73/−0.03
**C vs. Het**	34	0.352	0.689	0.390	0.003	0.44/−0.21
**C vs. Hom**	33	0.486	0.917	0.658	<0.0001	0.55/−0.33

C= control patients; Het = heterozygous for β-thalassemia patients; Hom = homozygous for β-thalassemia patients; N, number of samples; R^2^X, R^2^Y, values indicating the variance of the model; Q^2^, value indicating the predictive ability of the model. The *p*-value was considered significant for values <0.05.

## References

[1] Weatherall D.J., Clegg J.B. (2001). The Thalassemia Syndromes.

[2] Cao A. (1994). William Allan Award address. Am. J. Hum. Genet..

[3] Thein S.L. (2013). The molecular basis of β-thalassemia. Cold Spring Harb. Perspect. Med..

[4] Cao A., Rosatelli M.C., Monni G., Galanello R. (2002). Screening for thalassemia: A model of success. Obstet. Gynecol. Clin. N. Am..

[5] Monni G., Peddes C., Iuculano A., Ibba R.M. (2018). From Prenatal to Preimplantation Genetic Diagnosis of β-Thalassemia. Prevention Model in 8748 Cases: 40 Years of Single Center Experience. J. Clin. Med..

[6] Nagana Gowda G.A., Zhang S., Gu H., Asiago V., Shanaiah N., Raftery D. (2008). Metabolomics-based methods for early disease diagnostics. Expert Rev. Mol. Diagn..

[7] Cindrova-Davies T., Tissot van Patot M., Gardner L., Jauniaux E., Burton G.J., Charnock-Jones D.S. (2015). Energy status and HIF signalling in chorionic villi show no evidence of hypoxic stress during human early placental development. Mol. Hum. Reprod..

[8] Dunn W.B., Brown M., Worton S.A., Davies K., Jones R.L., Kell D.B., Heazell A.E.P. (2012). The metabolome of human placental tissue: Investigation of first trimester tissue and changes related to preeclampsia in late pregnancy. Metabolomics.

[9] Dettmer K., Aronov P.A., Hammock B.D. (2007). Mass spectrometry-based metabolomics. Mass Spectrom. Rev..

[10] Griffin J.L., Atherthon H., Shockcor J., Atzori L. (2011). Metabolomics as a tool for cardiac research. Nat. Rev. Cardiol..

[11] Poddighe S., Murgia F., Lorefice L., Liggi S., Cocco E., Marrosu M.G., Atzori L. (2017). Metabolomic analysis identifies altered metabolic pathways in Multiple Sclerosis. Int. J. Biochem. Cell Biol..

[12] Syggelou A., Iacovidou N., Atzori L., Xanthos T., Fanos V. (2012). Metabolomics in the developping human being. Pediatr. Clin. N. Am..

[13] De Sanctis V., Soliman A.T., Elsedfy H., Skordis N., Kattamis C., Angastiniotis M., Karimi M., Yassin M., El Awwa A., Stoeva I. (2003). Growth and endocrine disorders in thalassemia: The international network on endocrine complications in thalassemia (I-CET) position statement and guidelines. Indian J. Endocrinol. Metab..

[14] De Sanctis V., Soliman A.T., Elsedfy H., Pepe A., Kattamis C., El Kholy M., Yassin M. (2016). Diabetes and Glucose Metabolism in Thalassemia Major: An Update. Expert Rev. Hematol..

[15] Musharraf S.G., Iqbal A., Ansari S.H., Parveen S., Khan I.A., Siddiqui A.J. (2017). β-Thalassemia Patients Revealed a Significant Change of Untargeted Metabolites in Comparison to Healthy Individuals. Sci. Rep..

[16] Monni G., Ibba R.M., Lai R., Cau G., Mura S., Olla G., Rosatelli M.C., Cao A. (1993). Early transabdominal chorionic villus sampling in couples at high genetic risk. Am. J. Obstet. Gynecol..

[17] Monni G., Ibba R.M., Zoppi M.A., Milunsky A., Milunsky J.M. (2010). Prenatal Genetic Diagnosis through Chorionic Villus Sampling. Genetic Disorders and the Fetus.

[18] Murgia F., Iuculano A., Peddes C., Santoru M.L., Tronci L., Deiana M., Atzori L., Monni G. (2019). Metabolic Fingerprinting of Chorionic Villous Samples in Normal Pregnancy and Chromosomal Disorders. Prenat. Diagn..

[19] R Core Team (2011). R: A language and environment for statistical computing. R Foundation for Statistical Computing. http://www.R-project.org/.

[20] Smith C.A., Want E.J., O’Maille G., Abagyan R., Siuzdak G. (2006). XCMS: Processing mass spectrometry data for metabolite profiling using nonlinear peak alignment, matching, and identification. Anal. Chem..

[21] Liggi S., Hinz C., Hall Z., Santoru M.L., Poddighe S., Fjeldsted J., Atzori L., Griffin J.L. (2018). KniMet: A pipeline for the processing of chromatography-mass spectrometry metabolomics data. Metabolomics.

[22] Eriksson L., Byrne T., Johansson E., Trygg J., Wikström C. (2013). Multi- and Megavariate Data Analysis Basic Principles and Applications.

[23] Chong J., Soufan O., Li C., Caraus I., Li S., Bourque G., Wishart D.S., Xia J. (2018). MetaboAnalyst 4.0: Towards more transparent and integrative metabolomics analysis. Nucleic Acids Res..

[24] Iuculano A., Murgia F., Peddes C., Santoru M.L., Tronci L., Deiana M., Balsamo A., Euser A., Atzori L., Monni G. (2019). Metabolic characterization of amniotic fluids of fetuses with enlarged nuchal translucency. J. Perinat. Med..

[25] Wamelink M.M., Struys E.A., Jakobs C. (2008). The biochemistry, metabolism and inherited defects of the pentose phosphate pathway: A review. J. Inherit. Metab. Dis..

[26] Stincone A., Prigione A., Cramer T., Wamelink M., Campbell K., Cheung E., Olin-Sandoval V., Grüning N.M., Krüger A., Alam M.T. (2015). The return of metabolism: Biochemistryand physiology of the pentose phosphate pathway. Biol. Rev..

[27] Wood T. (1986). Physiological functions of the pentose phosphate pathway. Cell Biochem. Funct..

[28] Mahdi E.A. (2014). Relationship between oxidative stress and antioxidant status in beta thalassemia major patients. ActaChim. Pharm. Indica.

[29] Fibach E., Mutaz D. (2019). Oxidative Stress in β-Thalassemia. Mol. Diagn. Ther..

[30] Voskou S., Aslan M., Fanis P., Phylactides M., Kleanthous M. (2015). Oxidative stress in β-thalassaemia and sickle cell disease. Redox Biol..

[31] Pavlova L.E., Savov V.M., Petkov H.G., Charova I.P. (2007). Oxidative stress in patients with β-thalassemia major. Prilozi.

[32] Balboa M.A., Balsinde J. (2006). Oxidative stress and arachidonic acid mobilization. Biochim. Biophys. Acta.

[33] Sapirstein A., Bonventre J.V. (2000). Phospholipases A2 in ischemic and toxic brain injury. Neurochem. Res..

[34] Tallima H., El Ridi R. (2018). Arachidonic acid: Physiological roles and potential health benefits—A review. J. Adv. Res..

